# Substrate Stiffness Modulates Cell-Network Topology in Human-Derived Neurons

**DOI:** 10.1021/acsomega.5c12283

**Published:** 2026-04-02

**Authors:** Giulia Bruno, Giuseppina Iachetta, Luca Ceseracciu, Riccardo Carzino, Luigi Bruno, Julien Hurtaud, Francesco Gentile, Francesco De Angelis

**Affiliations:** † 121451 Italian Institute of Technology, Plasmon Nanotechnologies, via Morego 30, 16163 Genova, Italy; ‡ 9325 University Magna Graecia of Catanzaro, Department of Experimental and Clinical Medicine, Viale Europa, 88100 Catanzaro, Italy; § 18950 University of Calabria, Department of Mechanical, Energy, and Management Engineering, Via Bucci 44C, 87036 Rende (CS), Italy

## Abstract

Using confocal imaging and network topology analysis, we investigated how substrate stiffness affects adhesion and connectivity in hiPSC-derived neuronal cultures grown on polydimethylsiloxane (PDMS). We compared soft (∼12 kPa) and stiff (∼1.5 MPa) substrates with matched surface properties to isolate mechanical effects. Confocal analysis of NCAM expression revealed higher levels on soft PDMS at 10 DIV, indicating enhanced neuronal adhesion and outgrowth. Network topology analysis showed that only soft PDMS supported increased clustering, reduced path length, and higher small-worldness, reflecting more efficient connectivity. These results underscore the importance of substrate compliance in promoting neuronal development and inform scaffold design for neural engineering.

## Graphical abstract

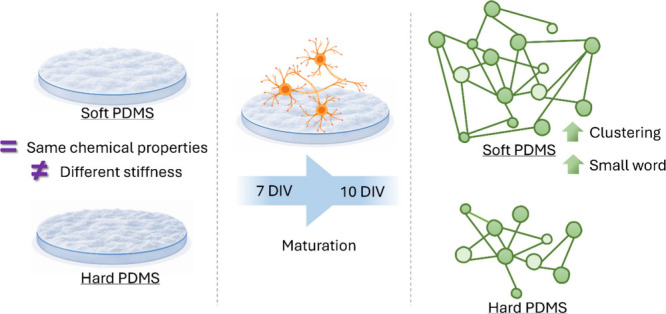

## Introduction

The mechanical properties of the cellular microenvironment critically regulate neuronal behavior,^1^ influencing processes such as differentiation,^2^ proliferation,^3^ migration, and network formation.^4,5^ In vivo, neurons develop within soft, viscoelastic tissues and are highly responsive to mechanical cues from the extracellular matrix.^6^ Understanding how neurons interpret these biomechanical signals is key for developing biomimetic platforms in neural tissue engineering and regenerative medicine.^7^

Substrate stiffness has been shown to affect cytoskeletal organization, morphology,^8^ synaptogenesis and electrophysiological activity,^9^ as well as gene expression.^10^ Recent studies, including work on brain organoids,^3^ demonstrate that tuning mechanical properties can alter developmental trajectories and regional specification, underscoring the role of mechanotransduction in brain development.^11^

Despite extensive research, how substrate stiffness influences the organization of human iPSC-derived neuronal networks remains incompletely understood. In this study, we examined neuronal growth and network formation on substrates with defined stiffness: soft PDMS (∼12 kPa), stiff PDMS (∼1.5 MPa), and glass as a rigid control. Substrates were mechanically characterized to ensure reliable comparisons. We used Neural Cell Adhesion Molecule (NCAM) immunostaining to probe neuronal attachment and early differentiation, as NCAM mediates neuron–substrate interactions and is sensitive to mechanical cues.^12,13^

To complement molecular data, we conducted a graph-based analysis of neuronal network topology across conditions, identifying small-world features that reflect efficient connectivity and modular structure.^14,15^ Our findings reveal that softer substrates promote stronger NCAM expression and more mature, clustered network organization, linking mechanical properties to both cell adhesion and higher-order neuronal patterning.

This integrative approach provides new insight into how mechanical cues regulate human neuronal development and supports the creation of more physiologically relevant in vitro models for neural tissue engineering and disease modeling.

## Results

### Mechanical Characterization

To investigate the effects of substrate stiffness on neuronal cell behavior, we cultured hiPSC-derived neurons on three different substrates: glass coverslips (control), and two Polydimethylsiloxane (PDMS) formulations, Sylgard 184 (hard) and Sylgard 527 (soft), which differ by orders of magnitude in stiffness. A schematic representation of the experimental timeline and culture conditions is shown in Figure 1A, while a detailed description of the experimental materials and methods is provided in the Methods section.

**Figure 1:**
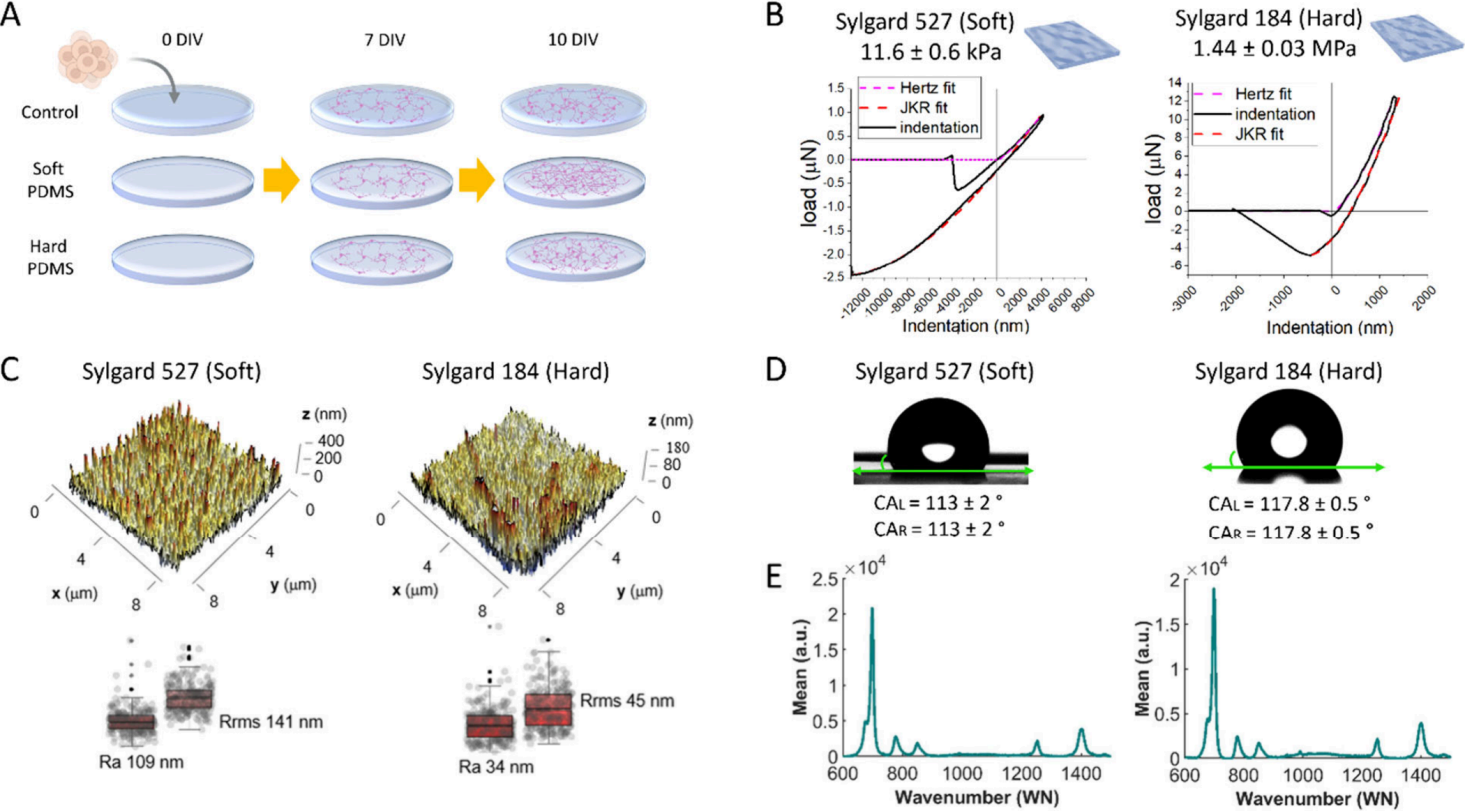
(A) Schematic of the experimental design with the three substrates: Control (top), Soft PDMS (Sylgard 527), and Hard PDMS (Sylgard 184). (B) Typical force–indentation curves of Sylgard 184 (right) and Sylgard 527 (left). The models fits are also shown, in red (JKR model) and in magenta (Hertz model). (C) Average roughness (Ra) and root-mean-square roughness (Rrms) values determined from morphological data. The values indicate that the PDMS surface is not flat at the nanoscale. (D) Contact angle measurements of Soft and Hard PDMS. (E) Raman measurements on Soft and Hard PDMS.

To characterize the substrates, we first measured their mechanical properties using a Chiaro nanoindenter (Optics11, Nederland), a cantilever-based instrument that uses calibrated probes and interferometric displacement recording to evaluate quantitatively mechanical properties of soft materials. The load–displacement curves were analyzed with the Johnson–Kendall–Roberts (JKR) contact model to take into account the adhesion of the material to the probe (Figure 1B). Sylgard 527 exhibited a Young’s modulus of 11.6 ± 0.6 kPa, while Sylgard 184 showed a significantly higher stiffness of 1.44 ± 0.03 MPa confirming the large differences in substrate stiffness. The importance of selecting the best model to interpret data can be seen in the comparison with the values obtained with the Hertz model, overestimated by 30–80% (20.8 kPa and 1.9 MPa for Sylgard 527 and Sylgard 184, respectively). The fitting curves are reported in Figure 1B, together with typical experimental curves, for clarity.

Topographical analysis by 3D profilometer^14^ revealed distinct nanoscale surface morphologies between the two PDMS types (Figure 1C). The soft PDMS (Sylgard 527) exhibited a rougher surface, with an average roughness (Ra) of 109 nm and root-mean-square roughness (Rrms) of 141 nm. In contrast, the hard PDMS (Sylgard 184) had a considerably smoother surface, with Ra and Rrms values of 34 and 45 nm, respectively.

Fourier transform analysis further enabled to determine the power spectrum (PS) density function of sample surfaces, reported in the separate Supporting Information Figure S1. By fitting data within the linear region of the plot, we determined fractal dimension (Df) values of 2.636 for the soft PDMS surface and 2.686 for the harder one. This minimal difference (<2%) indicates that both substrates exhibit similar scaling behavior. This similarity suggests comparable adhesive properties between the two PDMS surfaces, consistently with reports proposing fractal descriptors as more predictive for cell-adhesion behavior at the nanoscale.^16^

Contact angle measurements confirmed similar hydrophobicity for both PDMS surfaces, with Sylgard 527 at 113 ± 2° and Sylgard 184 slightly higher at 117.8 ± 0.5° (Figure 1D). Surface energy density, estimated via the Young–Dupree equation, was comparable in the two cases: ∼40 mJ/m^2^ for soft and ∼44 mJ/m^2^ for hard PDMS.

To complete the physio-chemical characterization, Raman spectroscopy was performed to verify the chemical identity and uniformity across the different substrates (Figure 1E). The spectral profiles of both PDMS formulations showed characteristic peaks corresponding to PDMS, confirming consistency across the substrates.

These findings suggest that, despite their mechanical contrast, the two PDMS substrates may present similar surface cues in terms of adhesion-related properties, making them valuable platforms for investigating how substrate stiffness and roughness alone influence biological responses.

### Confocal Imaging and NCAM Analysis

Following the characterization, we investigated how substrate stiffness influences neuronal adhesion, morphology, and maturation. For the scope, hiPSC-derived neurons (Ncardia) were cultured on glass coverslips (control), soft PDMS (Sylgard 527), and hard PDMS (Sylgard 184), all coated with 0.1% polyethylenimine (PEI). Glass served as a stiff control comparable to silicon substrates. All cultures were maintained under identical conditions to isolate the effect of substrate stiffness (Methods section).

To assess adhesion and morphology, we performed immunostaining for NCAM (neuronal cell adhesion molecule), MAP2 (microtubule-associated protein), and DAPI (nuclei). Confocal imaging (NIS Elements AR; Nikon) was performed at 7 and 10 DIV on three biological replicates per condition (two independent batches). In each sample, 5 to 8 high-resolution confocal images were acquired using a 60× objective with a 9 × 9 stitched field to ensure sufficient spatial coverage. Representative confocal images are shown in Figure 2A, displaying the three fluorescent channels: DAPI (blue), MAP2 (green), and NCAM (red), along with the corresponding merged images. The top panels correspond to 7 days in vitro (DIV), and the bottom panels to 10 DIV.

**Figure 2:**
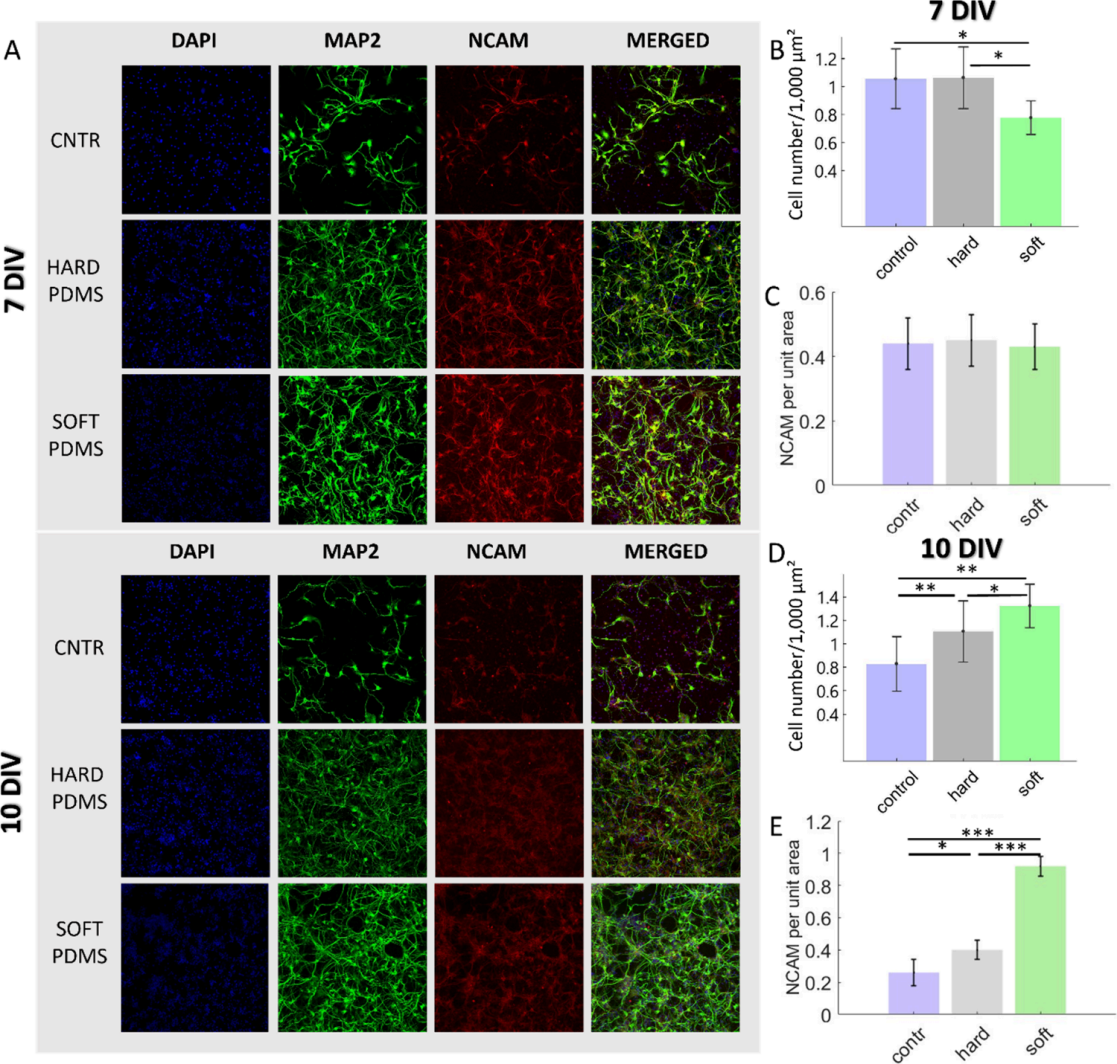
(A) Confocal images showing four channels: nuclei (DAPI, blue), neuronal dendrites (MAP2, green), neuronal cell adhesion molecule (NCAM, red), and the merged image. (B) Quantification of total cell number based on DAPI staining at 7 DIV. (C) Quantification of NCAM expression per unit area on the three substrates at 7 days in vitro (DIV). No statistically significant differences were observed, indicating homogeneous expression across conditions. (D) Quantification of total cell number based on DAPI staining at 10 DIV. (E) Quantification of NCAM expression per unit area on the three substrates at 10 DIV. *p*-values indicate statistical significance (*p* < 0.05, **p < 0.001).

At the early stage of culture (7 DIV), cell density shows no significant difference across substrates, with comparable values between the hard PDMS surface (1.00 ± 0.25 cells/1,000 μm^2^) and the control (1.06 ± 0.21 cells/1,000 μm^2^), while slightly lower values are observed on the soft PDMS surface (0.78 ± 0.12 cells/1,000 μm^2^) **(** Figure 2B). However, by 10 DIV, substrate-dependent differences in cell density become more pronounced. Notably, cultures on soft PDMS show a marked increase in cell density (1.10 ± 0.15 cells/1,000 μm^2^), compared to both hard PDMS (0.92 ± 0.25 cells/1,000 μm^2^) and the control (0.69 ± 0.19 cells/1,000 μm^2^), indicating a stiffness-dependent effect on cell retention and proliferation (Figure 2D).

NCAM expression was quantified as fluorescence intensity normalized by area. At 7 DIV, NCAM levels were comparable across all substrates (average 0.440 ± 0.044), with no significant differences (*p* > 0.5, one-way ANOVA) (Figure 2C), suggesting that, at early stages, neuronal adhesion and NCAM expression are primarily governed by early maturation processes rather than by substrate stiffness. This likely reflects the fact that hiPSC-derived neurons are still in the initial stages of adhesion and have not yet fully upregulated NCAM expression, which typically increases as neurons mature and establish stable networks.^17^ However, by 10 DIV, NCAM expression increased substantially, especially on soft PDMS (0.92 ± 0.06), compared to hard PDMS (0.40 ± 0.06) and glass (0.26 ± 0.08) (Figure 2E).

This upregulation is consistent with a more advanced stage of neuronal maturation and network organization observed on soft PDMS, suggesting that substrate compliance indirectly supports these processes. Softer PDMS may facilitate neuron–substrate interactions and cytoskeletal reorganization, creating conditions that favor neurite extension and the emergence of more complex neuronal networks.^18^ Overall, soft PDMS substrates provide a microenvironment that supports neuronal maturation and network organization at later stages of culture, with increased NCAM expression reflecting this more advanced organizational state.

### Topological Analysis of Neuronal Cell Networks on Soft and Hard Polymeric Surfaces

To further understand how substrate mechanical properties shape neuronal development, we examined the topology of the resulting networks at different culture stages. Figures 3D–F are a graph depiction of representative networks for the control (Figure 3D), soft (Figure 3E) and hard (Figure 3F) surfaces, measured at 10 DIV. Resulting prototypical networks are increasingly more packed, and exhibit increasingly more nodes and connections, moving from the control, to hard, to soft surfaces. We then quantitatively examined neuronal networks obtained from real biological cell cultures using networks science.^19,20,14^ Figures 3G–I report topological metrics of networks measured at 7 DIV, such as, the clustering coefficient (*cc*), the characteristic path length (*cpl*), the small world coefficient (*sw*) (Supporting Information S2).

**Figure 3:**
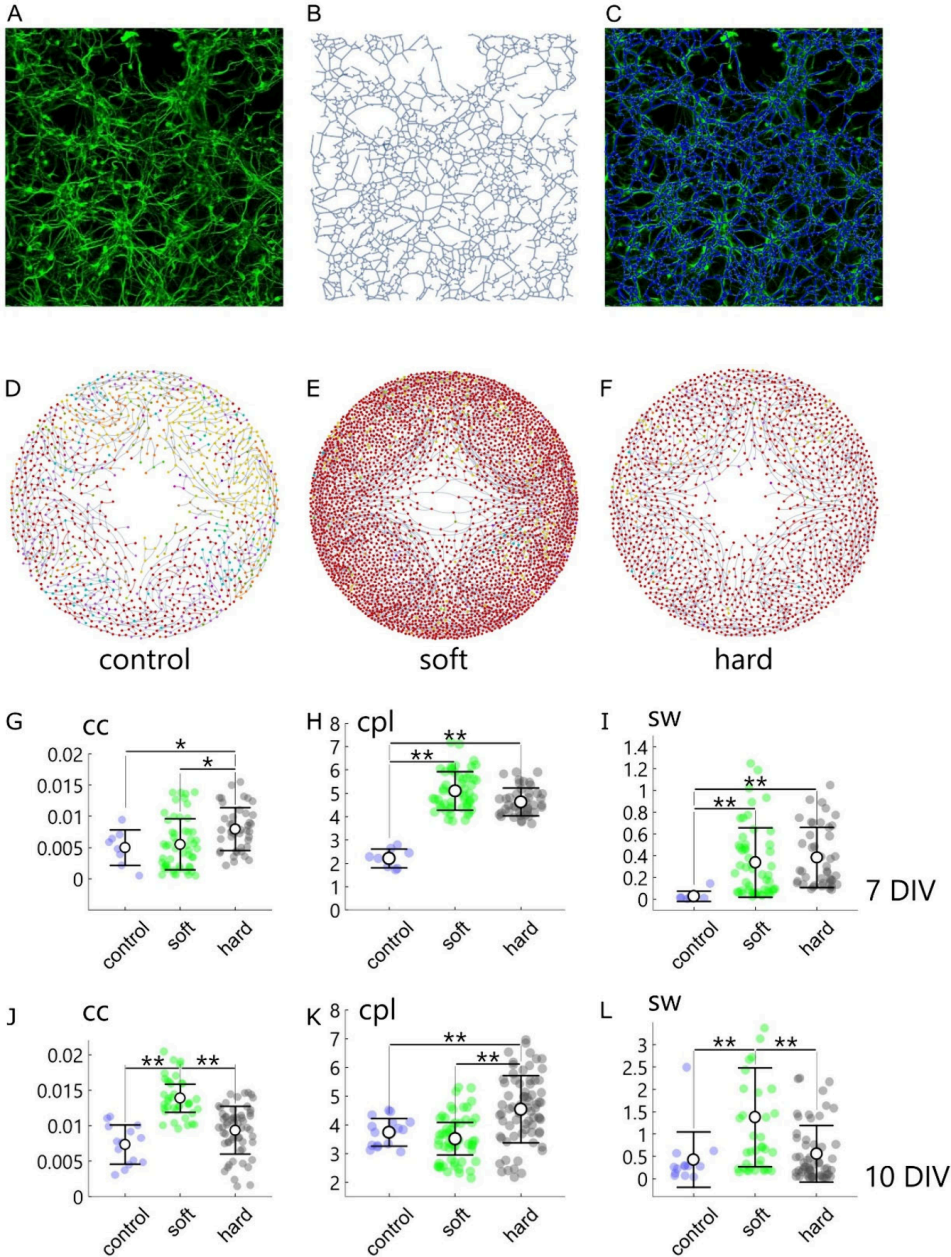
Topological cell analysis. Representative fluorescence image of neuronal dendrites adhering to soft PDMS surfaces, with dendrites labeled using the MAP2-specific marker (A). Network structure corresponding to dendrite distribution was identified using image analysis algorithms described in the Methods section (B). Merged image of fluorescence micrograph of neuronal cells and network structure (C). Graph representations of neuronal networks on control (D), soft (E), and hard (F) surfaces at 10 DIV, visualized using a gravity-based layout algorithm that arranges vertices by minimizing mechanical energy to intuitively reflect network structure. Quantitative metrics of the networks at 7 DIV: clustering coefficient (cc) (G), characteristic path length (cpl) (H), and small-world coefficient (sw) (I). Same metrics measured at 10 DIV: cc (J), cpl (K), and sw (L).

Results of the analysis illustrate that, at 7 DIV, the average value of the clustering coefficient does not vary significantly moving from the control (*cc*∼0.0049) to soft surfaces (*cc*∼0.0055). In contrast, the clustering coefficient measured on hard surfaces (*cc*∼0.0079) is significantly different from that observed on soft surfaces (*p* < 0.005) and the control (*p* < 0.05) (Figure 3G). Regarding the characteristic-path-length, the value determined for the control (*cpl*∼2.21) is significantly lower than that measured on both soft (*cpl*∼5.1) and hard surfaces (*cpl*∼4.62), with *p* < 0.001 (Figure 3H). The values of the small-world coefficient are consistent with the previously observed trends: the control condition shows the lowest value (*sw*∼0.0268), followed by neuronal networks on soft (*sw*∼0.34) and hard surfaces (*sw*∼0.38). While the small world coefficient varies significantly from the control to either soft or hard surfaces (*p* < 0.001), it does not show significant differences between the latter two configurations (Figure 3I). Thus, analysis of neuronal networks after 7 days from culture indicates that PDMS substrates, either soft or hard, induce significant cell assembly compared to the control, evidenced by larger values of *sw*. Differently, neuronal networks behave approximately in the same way on PDMS surfaces, regardless of their elasticity.

Moving to 10 DIV, one can observe that the average *cc* of networks on soft surfaces (*cc*∼0.014) is larger compared to the same metrics computed for hard surfaces (*cc*∼0.0094), and the control (*cc*∼0.0073). The difference is significant between the soft and the hard substrate (*p* < 0.001), and between the soft surface and the control (*p* < 0.001) (Figure 3J). Instead, the difference is not significant between the hard surface and the control (*p* > 0.05). When examining the characteristic path length, we found values of approximately *cpl*∼3.75 for the control, *cpl*∼3.52 for soft surfaces, and *cpl*∼4.54 for hard surfaces (Figure 3K). For this metric, soft surfaces - showing the lowest *cpl* -differ significantly from hard surfaces (*p* < 0.001), but not from the control (*p* > 0.05). Values of small-world coefficient, i.e. a combination of *cc* and *cpl*, illustrate that on soft surfaces neuronal cells assemble into networks with enhanced topological characteristics (*sw*∼1.37) compared to the control (*sw*∼0.43) and, remarkably, to hard surfaces (*sw*∼0.56). The difference is statistically significant between soft and hard surfaces, and between soft surfaces and the control, with significance levels *p* < 0.001 in both cases (Figure 3L). The difference is not statistically significant in all other cases (*p* > 0.05).

Collectively, results illustrate that at the early stage of network formation (7 DIV), neuronal cells on polymeric surfaces exhibit values of small world coefficient larger than the control, suggesting more efficient layouts and enhanced performance of the system in terms of transport of information, nutrients, and signals. For this time point, the soft and hard surface exhibit similar behavior. Differently, at the late stage of network formation (10 DIV), neuronal cells exhibit the highest level of organization on soft polymeric surfaces, evidenced by values of small-world-ness significantly higher than on control and, notably, hard surfaces. In addition to this, a qualitative topological analysis of neuronal networks is provided in Supporting Information, Section S3. In this analysis, networks were reconstructed from nuclear positions using a distance-based rule (Waxman model), which offers a simplified and complementary representation of network organization. Consistently with the main network reconstruction approach based on neurite morphology, the trends observed in the topological parameters derived from the Waxman model qualitatively align with the increased NCAM expression observed at 10 DIV on soft substrates, supporting the interpretation of a more advanced network organization rather than implying a direct causal relationship.

## Discussion

This study demonstrates that substrate stiffness plays a critical role in modulating neuronal adhesion and network development in hiPSC-derived neuronal cultures. By using well-characterized PDMS substrates (soft PDMS (Sylgard 527, 11.6 ± 0.6 kPa), hard PDMS (Sylgard 184, 1.44 ± 0.03 MPa), and glass with comparable surface chemistry and wettability, we aimed to minimize physicochemical confounding factors and focus on the contribution of mechanical properties to neuronal behavior at both the cellular and network levels.

Despite their markedly different elastic moduli, Sylgard 527 and Sylgard 184 exhibit similar surface hydrophobicity, chemical composition, and fractal-like scaling behavior. While the two PDMS formulations differ in average roughness, their comparable fractal dimension indicates similar multiscale surface organization, which has been reported to be more relevant than roughness alone for cell–surface interactions. This detailed characterization supports the interpretation that differences in neuronal behavior primarily reflect the influence of substrate compliance rather than unintended variations in surface chemistry or topography. As such, this substrate system provides a controlled framework to examine how mechanical cues contribute to neuronal organization when combined with network topology analysis.

Our findings show that early stage neuronal adhesion (7 DIV) is largely unaffected by stiffness, as indicated by similar NCAM expression across all substrates. However, by 10 DIV, soft PDMS significantly enhanced NCAM expression, suggesting increased neuron–substrate interactions and cytoskeletal organization that support more robust neurite outgrowth. adhesion molecule dynamics during the transition from early attachment to network maturation.

Complementary network topology analysis revealed that both soft and hard PDMS substrates promoted early network formation at 7 DIV, as shown by higher clustering and small-world coefficients compared to the glass control. This suggests that PDMS materials, regardless of stiffness, support initial network assembly. However, by 10 DIV, only the soft PDMS substrate maintained significant enhancements across all key parameters, such as higher clustering, reduced characteristic path length, and the highest small-world coefficient, indicating more efficient organization. In contrast, networks on glass remained more spatially clustered and less interconnected, reflecting limited capacity for topological reorganization. The emergence of small-world topological features in neuronal cultures grown on compliant substrates should be interpreted in the context of structural and developmental organization rather than as direct evidence of mature functional connectivity. In developing in vitro neuronal systems, small-world metrics primarily reflect the balance between local clustering of neurites and global spatial integration of the network, capturing how cells self-organize in response to mechanical cues. In this study, increased small-world-ness on soft PDMS surfaces (particularly at 10 DIV) indicates a more coordinated morphological arrangement characterized by enhanced clustering and reduced path length, consistent with stabilized neurite outgrowth and increased cell–cell interactions. Importantly, while small-world architectures are a hallmark of functional brain networks, their presence here should be viewed as an indicator of structural readiness and network maturation potential, rather than confirmed electrophysiological efficiency. The absence of direct functional measurements, such as multielectrode array recordings or calcium imaging, precludes conclusions regarding synaptic transmission or information processing. Instead, our results demonstrate that substrate compliance modulates the topological framework within which functional connectivity may later emerge, providing a mechanically driven bias toward architectures known to support efficient signaling in mature neural systems.

Collective cell effects, including cell–cell interactions, local density, and mechanical coupling, are likely to contribute to both individual neuronal behavior and emergent network architecture in our system. Substrate compliance does not act solely at the single-cell level, but modulates how neurons mechanically and biochemically interact with one another during network formation. On softer PDMS, enhanced substrate deformability may facilitate force transmission across cell–cell contacts, promoting coordinated neurite outgrowth and stabilization of intercellular connections, consistent with the increased NCAM expression observed at 10 DIV. Although local density may amplify contact-mediated signaling, density alone cannot account for the observed network topology, as stiffness-dependent differences emerge even when cell densities are comparable and are not recapitulated by higher density on stiffer substrates. These findings suggest that substrate mechanics regulate the integration of collective interactions rather than simply increasing contact probability. More broadly, they raise the possibility that compliant microenvironments enable neurons to enter a mechanically coupled regime in which local interactions are amplified into global network organization: a hypothesis that warrants direct investigation through future studies combining mechanical perturbations, traction-force measurements, and live imaging of network assembly.

## Conclusion

Together, these results highlight the importance of substrate compliance not only for improving in vitro neural models for developmental studies and disease modeling but also for advancing our understanding of how mechanical cues shape neuronal connectivity. Beyond translational relevance, the integrated analysis of mechanical properties, adhesion molecule expression, and emergent network topology also provides valuable insights for basic neuroscience, particularly in the context of neuronal development and mechanobiology. Understanding how physical cues such as stiffness influence neuronal adhesion, connectivity, and maturation can shed light on the biophysical forces shaping neural circuit formation in vivo. While the present study employs substrates with macroscopically uniform stiffness, it is important to note that neurons in vivo experience spatially heterogeneous mechanical cues, including local stiffness gradients, which may further modulate neuronal adhesion, polarization, and network connectivity and represent an important direction for future investigation to further dissect the role of mechanical heterogeneity in neuronal network development.

A limitation of this study is that network topology was inferred from morphological and spatial connectivity rather than from direct measurements of neuronal activity. While small-world metrics provide valuable insight into the structural organization and developmental progression of neuronal assemblies, they do not directly report on synaptic efficacy, firing dynamics, or information flow. Consequently, the observed increase in small-world-ness (particularly on soft substrates) should be interpreted as reflecting enhanced structural coordination and network maturation potential, rather than confirmed functional connectivity. Future studies integrating electrophysiological approaches, such as multielectrode array recordings or calcium imaging, will be necessary to establish how mechanically driven changes in network topology translate into physiological activity and signal processing.

However, these findings contribute to a deeper comprehension of how mechanical properties interact with molecular programs during brain development and may inform future studies aimed at dissecting the role of mechanotransduction pathways in neurodevelopmental processes and disorders.

## Methods

### Fabrication of Polydimethylsiloxane Substrates with Tunable Mechanical Properties

Sylgard 184 (Dow Corning) was prepared by mixing the elastomer base with the curing agent at a 10:1 (w/w) ratio. The mixture was degassed under vacuum to remove air bubbles and subsequently drop-casted onto the cleaned glass coverslips. The coated coverslips were then cured in a ventilated oven at 65 °C for 20 min. Once cured, the PDMS-coated glass coverslips were placed into 35 mm Petri dishes for cell culture.

Sylgard 527 (Dow Corning) was prepared by mixing components A and B at a 1:1 (w/w) ratio. The mixture was degassed under vacuum and deposited onto glass coverslips. The substrates were then cured overnight at 65 °C. After curing, the soft PDMS-coated coverslips were also transferred to 35 mm Petri dishes for subsequent use in cell culture.

Control samples consisted of cleaned glass coverslips without PDMS coating, which were directly placed into Petri dishes for culture.

### Mechanical Characterization

#### Stiffness Measurements

Mechanical characterization was performed using a Chiaro bioindenter (Optics11), optimized for soft materials. The system uses a sphere-tipped cantilever and an interferometer to measure force during indentation. A piezo tube displaces the probe, and the resulting cantilever deflection yields the reaction force, while indentation depth is calculated from the imposed displacement. Tests were performed in demineralized water, in displacement mode with a 8 mm radius, 3.65 N/m stiffness probe, chosen to be suitable for both materials. After testing, the elastic modulus was calculated by fitting the load-indentation curve with the chosen model. In our case, specifically, we applied the Johnson–Kendall–Roberts model, preferred to the more common Hertzian model to avoid overestimation of the modulus arising from the modification of the contact area caused by adhesion; this model has shown higher reliability and lower dependence on the probe size than the Hertz model.^21,22^ Fifteen indentations were performed on each material, results are presented as average and standard deviation. Only curves fitted with a coefficient of determination R2 > 0.95 were considered for further analysis.

#### 3D Profilometer

Topographical images of soft and hard PDMS surfaces were obtained using a Tribometer MFT-5000 equipped with an integrated 3D profilometer from Rtec, following the procedure detailed in reference.^14^ The profilometer, fitted with 50× and 100× objectives, operated in noncontact confocal mode under white light illumination to generate 3D interferometric surface profiles. Vertical scans along the *z*-axis were performed to capture the surface topography. Each scan was conducted with a step size of 2.5 nm and an acquisition rate of 7 frames per second. The resulting images measured 1280 pixels in width by 960 pixels in height, with an approximate pixel size of 78 nm, covering a total imaged area of 100 × 75 μm per sample. For each combination of PDMS-to-curing-agent ratio and sample stiffness, at least five measurements were conducted.

#### Water Contact Angle

Wettability measurements were performed with the sessile drop method using a DataPhysics OCAH 200 contact angle goniometer under laboratory conditions (temperature 22–25 °C and relative humidity 50–60%). For the characterization, droplets of 5 μL volume Milli-Q water were used.

#### Raman Spectroscopy Measurements

Raman spectroscopy was employed to assess the chemical composition of the substrates. Measurements were conducted using a confocal Raman microscope equipped with a 633 nm excitation laser set to a power of 5 mW at the sample plane. Spectra were acquired with an integration time of 10 s per point and averaged across five randomly selected locations on each substrate to ensure representative sampling. To improve spectral quality and consistency, standard preprocessing steps were applied, including baseline subtraction and cosmic ray removal.

### Cell Culture

Human induced pluripotent stem cell (iPSC)-derived neurons (CNS.4U) were purchased from Ncardia (Gosselies, Belgium). The samples were previously sterilized by ultraviolet (UV) exposure for 30 min and subsequently coated with a 0.1% polyethylenimine (PEI) solution diluted in borate buffer for 1 h at room temperature. The neurons were plated directly onto PDMS-coated glass coverslips and control coverslips at a density of 80,000 cells per sample and cultured according to the manufacturer’s specifications. Immunofluorescence staining was performed at 7 and 10 days postplating.

### Immunofluorescence Staining

Cells were fixed with a 4% (w/v) paraformaldehyde solution for 15 min, permeabilized with 0.1% Triton X-100 for 15 min followed by washing with phosphate buffered saline (PBS) twice. Blocking was performed using 3% bovine serum albumin (BSA) for 30 min followed by incubation with MAP2 (rabbit monoclonal, Thermo Fisher Scientific) and NCAM (mouse monoclonal, Thermo Fisher Scientific) antibodies diluted 1:100 in a solution of 1% BSA for 2 h at room temperature. After three washes in PBS, cells were incubated with Alexa Fluor 488 goat antirabbit and Alexa Fluor 647 goat antimouse secondary antibodies (diluted 1:500 in 1% BSA) for 1 h at room temperature, followed by three washes with PBS. Finally, cells were incubated for 5 min with 4′,6-diamidino-2-phenylindole (DAPI) to stain the nuclei and then visualized under the confocal microscope (NIS Elements AR; Nikon) with appropriate filter sets for Alexa Fluor 488 (Ex/Em 495/519 nm), Alexa Fluor 647 (Ex/Em 650/668 nm), and DAPI (Ex/Em 358/461 nm).

#### Quantitative Image Analysis

Quantitative analysis of NCAM expression was performed using high-resolution confocal images acquired at 7 and 10 days in vitro (DIV). Images were collected using a 60× objective with a 9 × 9 tile scan to ensure consistent sampling across conditions. Image processing and analysis were carried out using ImageJ. Background fluorescence was subtracted, and the NCAM signal (red channel) was isolated using consistent thresholding parameters across all samples. The integrated fluorescence intensity of the NCAM signal was measured and normalized to the corresponding imaged area, yielding a value of NCAM intensity per unit area. A minimum of 5–8 fields of view were analyzed per sample, with three biological replicates for each substrate condition.

### Cell-Network Analysis

We used MAP2 fluorescence images to determine the topological characteristics of neuronal networks on soft and rigid PDMS surfaces. In particular, we used the information about the distribution of MAP2 on different substrates, to determine a faithful representation of neuronal cell networks developing on the surfaces over time. To do this, we used the methods already implemented in reference.^14^

For each considered surface characteristics (soft and hard) and time (7 DIV and 10 DIV), and for the control, we examined green-fluorescent images of neuronal cells lining the PDMS surface). The images were first converted to grayscale and enhanced using a bilateral filter. Following this correction, a skeleton transformation was applied to reduce the foreground regions while preserving the original structure’s extent and connectivity, effectively removing most of the original foreground pixels. Smaller, disconnected objects were then removed. From the resulting skeleton, the morphological graph of the cells was extracted, identifying key branch points and end points.

## Supplementary Material



## Notes

Power spectrum analysis of PDMS substrate surfaces; detailed description and interpretation of topological network metrics (clustering coefficient, characteristic path length, and small-world coefficient); analysis of the small-world properties of cell networks on PDMS substrates computed via cell-nuclei routing (PDF)
