# Supplementary material for: Substrate Stiffness Modulates Cell-Network Topology in Human-Derived Neurons
Source: ACS Omega. 2026 Apr 2;11(14):21870–7. doi: 10.1021/acsomega.5c12283 (PMC13084456; doi:10.1021/acsomega.5c12283)
Supplement: Supplementary file 1 [file ao5c12283_si_001.pdf]

Supporting Information

Title: “Substrate Stiffness modulates cell-network topology in human-derived neurons”

Authors:

Giulia Bruno*[1], Giuseppina Iachetta[1], Luca Ceseracciu[1], Riccardo Carzino[1], Luigi Bruno[3], Julien Hurtaud[1], Francesco Gentile*[2], Francesco De Angelis[1]

Affiliation:

1. *Italian Institute of Technology, Plasmon Nanotechnologies, via Morego 30, 16163, Genova, Italy*
2. *University Magna Graecia of Catanzaro, Department of Experimental and Clinical Medicine, Viale Europa, 88100, Catanzaro, Italy*
3. *University of Calabria, Department of mechanical, energy, and management engineering, Via Bucci 44C, 87036, Rende (CS), Italy*

Contents

Supporting Information 1. Power spectrum of PDMS surface 2

Supporting Information 2. Description and significance of topological networks measures: clustering coefficient, characteristic path length, small-world coefficient 3

Supporting Information 3. The small-world-ness of cell-networks on PDMS surfaces computed by routing cell-nuclei 6

References 9

Supporting Information 1. Power spectrum of PDMS surface

The PS describes how the logarithms of the information content of a surface (Q) and of the scale (q) are correlated. The rate of change of Q with q, β, is in turn associated with the fractal dimension Df of the surface [1]. The fractal dimension is a measure of the complexity of a surface determined over hierarchical length scales. By fitting data within the linear region of the plot, we determined Df values of 2.636 for the soft PDMS surface and 2.686 for the harder one. The close similarity - less than 2% variation - indicates that both surfaces exhibit comparable scaling behavior. This, in turn, suggests that these surfaces may have similar adhesive properties, as indicated by a number of report that have attempted to introduce descriptors other than simple roughness to correctly describe the multifaceted nature of nanoscale surfaces, especially in the context of cell-adhesion [1].

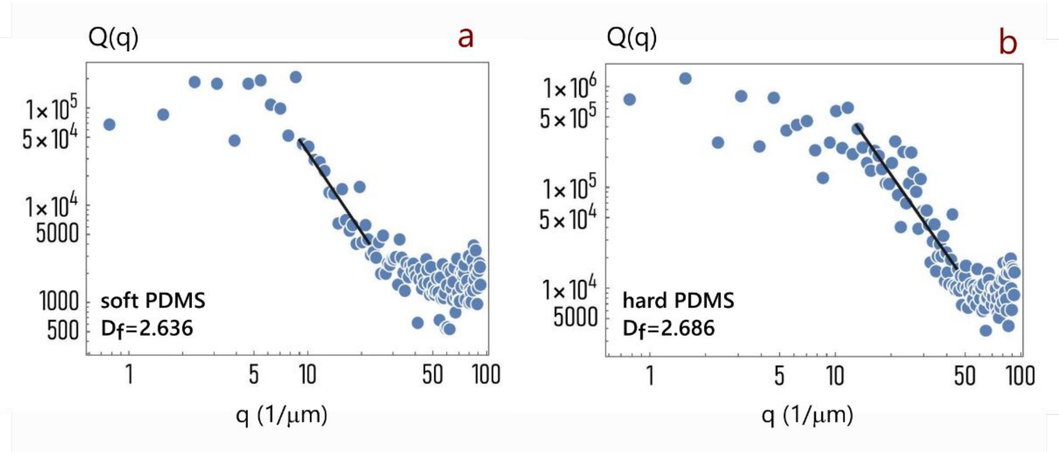

Supporting Information Figure S1. *Power spectrum of PDMS sample surfaces*. Power spectrum calculated for the soft (a) and hard (b) PDMS surface, showing the information content Q of the topographical image of samples as a function of space frequency q.

Supporting Information 2. Description and significance of topological networks measures: clustering coefficient, characteristic path length, small-world coefficient

S2.1 Description

Clustering Coefficient (cc). The clustering coefficient quantifies the degree to which nodes in a network tend to cluster together [2]. For a given node, it is the ratio of the number of existing links between its neighbors to the number of all possible links between them. Formally, for a node i with k_i neighbors, the local clustering coefficient is :

$$cc = \frac{2\, e_i}{k_i(k_i - 1)}$$

where e_i is the number of edges between the neighbors of node i. The average clustering coefficient cc is the mean of cc over all nodes.

Characteristic Path Length (cpl). The characteristic path length is the average shortest path between all pairs of nodes in the network. It reflects how efficiently information or signals can propagate across the network. Mathematically:

$$cpl = \frac{1}{N(N-1)} \sum_{i \neq j} d_{ij}$$

where d_{ij} is the shortest path length between nodes i and j, and N is the total number of nodes.

Small-World Coefficient (sw). The small-world coefficient evaluates how small-world a network is, typically using [2-4]:

$$sw = \frac{cc/cc_{rand}}{cpl/cpl_{rand}}$$

where cc and cpl are the clustering coefficient and path length of the real network, and cc_{rand} and cpl_{rand} are the same measures for a comparable random network. A network is considered small-world if $sw > 1$.

S2.2 Biological Significance

Understanding complex biological systems often involves analyzing them as networks, whether these are neural circuits in the brain, gene regulatory networks, protein interaction networks, or metabolic pathways. The topological features of these networks provide insights into their structure, function, and evolution. Below is a detailed explanation of the biological significance of clustering coefficient, characteristic path length, and small-world coefficient.

S2.2.1 ***Clustering Coefficient***. In biological networks, a high clustering coefficient reflects local redundancy and modularity. This means that nodes (neurons, proteins, or genes) that are connected to a common node are also likely to be interconnected among themselves. In particular:

Neuroscience: In the human brain, local clustering is associated with functional specialization. Cortical areas involved in similar functions often form tightly connected groups, enabling efficient local processing of sensory or cognitive information. For example, visual processing areas in the occipital cortex form dense local clusters.

Proteomics: In protein–protein interaction (PPI) networks, high clustering often corresponds to protein complexes or functional modules, where a group of proteins work together to perform a specific biological function, such as DNA replication or cell signaling.

Genomics: In gene regulatory networks, clusters of co-regulated genes often indicate a coherent functional response, like the activation of a metabolic pathway in response to environmental stimuli.

High clustering thus points to functional robustness, allowing localized damage (e.g., mutations or neural lesions) to have limited global impact.

S2.2.2 ***Characteristic Path Length***. The characteristic path length describes the average communication distance in a network. In biological terms, it reflects how quickly and efficiently information can propagate through the system. Specifically, in the following fields:

Neural Systems: A shorter path length facilitates fast signal transmission across brain regions, essential for integrative cognitive tasks such as decision-making, memory retrieval, and consciousness. Changes in path length have been linked to neurodegenerative diseases: for instance, Alzheimer's disease is associated with increased path lengths, reflecting disrupted global communication.

Metabolic Networks: In cellular metabolism, a short path length implies that metabolites can be converted into each other with minimal intermediate steps, increasing metabolic efficiency and adaptability.

Ecosystems and Food Webs: Even in ecological networks, short path lengths indicate resilient and well-connected systems, where energy or nutrient flow can be re-routed in the face of species loss or environmental stress.

In general, a low characteristic path length supports efficient and resilient information or resource transfer, which is vital for biological fitness.

S2.2.3 ***Small-World Coefficient***. The small-world coefficient combines the benefits of both high local clustering and short global path length, capturing a key organizational principle in biological systems.

Brain Networks: The small-world organization has been observed in structural and functional brain networks across species. This architecture allows for simultaneous segregation and integration-regions can process information locally while maintaining global coordination. It is thought to underpin complex behaviors and consciousness.

Gene and Protein Networks: Small-world topology in regulatory and interaction networks ensures that functional modules are tightly knit, yet remain accessible from the rest of the network, facilitating dynamic reconfiguration during development, stress responses, or disease states.

Evolutionary Advantage: Small-world topology is believed to be an evolutionarily favored trait. It provides a balance between wiring cost (e.g., axonal length or protein interaction maintenance) and efficiency, leading to networks that are both cost-effective and functionally powerful.

Disease and Disorders: Deviations from small-world properties can indicate dysfunction. For example, schizophrenia and epilepsy have been associated with disrupted small-world architecture in brain networks, suggesting that maintaining this balance is critical for healthy function.

S2.3 Summary

Topological metrics like clustering coefficient, path length, and small-world-ness offer a powerful lens to analyze the architecture, dynamics, and health of biological systems. They reveal how nature organizes complexity, through modularity, efficiency, and robustness, to support life's remarkable adaptability. By studying these network properties, researchers can better understand the principles underlying biological organization, detect early signs of dysfunction, and design interventions in medicine, synthetic biology, and neuroscience.

S2.4 Small-World Networks in Tissue Engineering and Cell Organization

The small-world topology - characterized by high local clustering and short global path lengths - has emerged as a key organizing principle in biological systems, from neuronal networks to multicellular assemblies. Recent studies have extended this concept to engineered tissues and in vitro cellular systems, where small-world-ness plays a crucial role in shaping functional connectivity, information processing, and collective behavior.

S2.4.1 ***Functional Enhancement in Engineered Cell Networks***. In neuronal cultures grown on 2D and 3D substrates, small-world architectures were shown to maximize the efficiency of information transfer, particularly at an optimal small-world coefficient around 4.8 ± 1 [5]. Gentile [6] further demonstrated that networks exhibiting this topology support enhanced signal fidelity and robustness, mimicking the structural-functional balance observed in the brain. Such findings underscore the utility of small-world metrics as functional readouts in tissue-like cellular systems.

S2.4.2 ***Influence of Nano-Topography on Cell Organization***. A consistent theme across multiple works (e.g., [7-10]) is the influence of nano-topographical cues in guiding cell self-organization into small-world networks. Substrates with multi-scale roughness and mesoporous architectures not only promote adhesion but also bias cells toward forming modular but globally connected clusters, enhancing both communication and functional specialization within the construct. These structural properties are crucial in designing biomimetic materials for neural repair or cancer modeling.

S2.4.3 ***Mechanical Constraints and Aggregate Formation***. Mechanical forces also play a pivotal role in shaping network topology. In Marinaro et al. [11], neuronal adhesion and clustering were modulated by the elasticity of the substrate, affecting how cells connect and distribute spatially. Gentile [12] proposed that the maximum size of cell aggregates is governed by a competition between cell–cell binding energies and mechanical strain energy, suggesting that small-world-like patterns emerge as a mechanical and energetic equilibrium state.

S2.4.4 ***Diagnostic and Predictive Value in Oncology***. Intriguingly, small-world metrics have diagnostic value. In studies on cancer cell responses to radiation [7,8,10], changes in network topology - particularly reductions in clustering or global efficiency—were associated with cellular damage, resistance, or death. This suggests that topological analysis can reveal hidden dimensions of cellular state, offering a non-invasive biomarker for treatment efficacy or disease progression.

Supporting Information 3. The small-world-ness of cell-networks on PDMS surfaces computed by routing cell-nuclei

Here we expound on a second method used to evaluate the small-world coefficient of networks of neuronal cells cultured on soft and hard surfaces, at different time frames, i.e. 7 DIV and 10 DIV. The method computes key topological features of cellular systems by examining networks built from the position of cell-centers on a surface, as described in a number of past works [7-9,13]. Specifically, the method uses DAPI fluorescence images of cells (**Supporting Information Figure S3a**), acquired using the methods reported in a separate section of the paper. DAPI fluorescence images of cells are then processed and Watershed transformed (**Supporting Information Figure S3b**), this enables to determine the position of the cells on the substrate. Cell-nuclei are then connected using a distance based rule, reported for the first time in reference [14], and known as the Waxman model (**Supporting Information Figure S3c**). The Waxman model makes a decision on whether two cells (i), (j) are connected on the basis their Euclidean distance d_{ij}, such that if

$$\alpha\, exp\left(-\, d_{ij}/\beta\, l\right) < p$$

there is link between i and j. In the equation above, α and β are model parameters, l is the maximum measured distance between cells of the system, and p is a threshold value. For a fixed α and β, the larger p the higher the number of interconnected points in the network. Then, the network resulting from the wiring if individual cell nuclei is evaluated by means of the topological parameters described in the **Supporting Information section 2**, i.e. the clustering coefficient, the characteristic path length, the small-world-coefficient. The diagrams in the **Supporting Information Figure S3d** and **Supporting Information Figure S3e** report the values of sw evaluated on soft and hard PDMS surfaces, and the control, 7 (**S3d**) and 10 (**S3e**) days from culture. We used, for these measurements, the following values of the model parameters: $\alpha = 1, \beta = 0.025, p = 0.8$. Diagrams illustrate that at the early stage of network formation (7 DIV), the small world coefficient on soft surfaces is smaller compared to hard surfaces, and the control. Being $sw{\sim}0.40$ for the soft, and $sw{\sim}0.48$ for the hard surfaces and the control. Differently, at the late stage of network formation (10 DIV) the small world coefficient is optimized on soft surfaces with a value $sw{\sim}0.91$, compared to $sw{\sim}0.84$ found on hard surfaces, and $sw{\sim}0.77$ for the control. These results qualitatively align with those obtained performing direct network reconstruction, and reported in the main text. Both demonstrate that, at steady state, soft PDMS surfaces promote the clustering of neuronal cells into structured networks, as indicated by higher small-world-ness values compared to hard surfaces and the control.

Additionally, because the results of the Waxman model depend on its parameters, we conducted an extensive testing campaign to assess the robustness of the analysis against variations in those parameters. Specifically, we verified the effect of changing the value of the probability threshold p in $0.65 - 0.9$ range, while keeping constant α and β as $\alpha = 1$, $\beta = 0.025$. Diagrams in the **Supporting Information Figure S3f** and **g** illustrate how values of small-world-ness vary as a function of p, for networks reconstructed 7 days (**S3f**) and 10 days (**S3g**) from culture. The results of this extended test campaign suggest that the cell behavior observed at $p = 0.8$ remains consistent across the full range of p values. In the 10 DIV configuration, cells on soft surfaces consistently exhibit higher small-world-ness coefficients compared to those on hard surfaces and the control, with the difference progressively increasing as p increases.

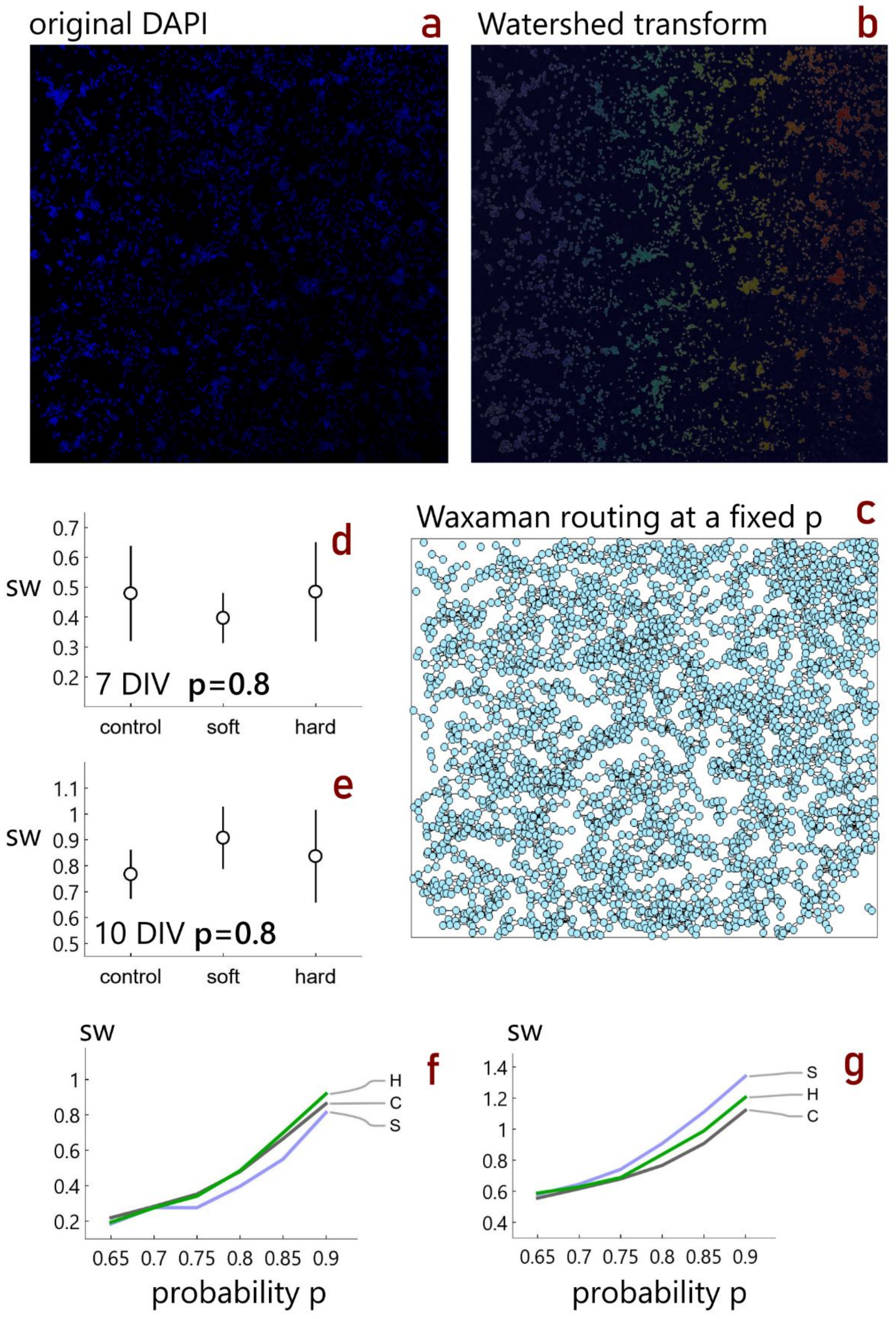

Supporting Information Figure S3.Small-world analysis of neuronal cell networks reconstructed from cell-nuclei positions on PDMS substrates. (a) Representative original DAPI fluorescence image used to identify cell nuclei. (b) Watershed-transformed image enabling segmentation and localization of individual nuclei. (c) Example of network reconstruction obtained by connecting nuclei according to the Waxman distance-based routing model. (d,e) Small-world coefficient (sw) of reconstructed networks on control, soft, and hard substrates at 7 DIV (d) and 10 DIV (e) using fixed model parameters ($\alpha = 1$, $\beta = 0.025$, $p = 0.8$). At 7 DIV, soft substrates show lower small-world-ness than hard substrates and control, whereas at 10 DIV soft substrates display the highest small-world coefficient. (f,g) Robustness analysis of the small-world

coefficient as a function of the Waxman probability threshold p (0.65–0.9) for networks reconstructed at 7 DIV (f) and 10 DIV (g). The trend is preserved across the explored range of p, with soft substrates consistently yielding higher small-world-ness at 10 DIV.

References

[1] Gentile, F., Tirinato, L., Battista, E., Causa, F., Liberale, C., di Fabrizio, E. M. & Decuzzi, P. 2010 Cells preferentially grow on rough substrates. *Biomaterials.* **31**, 7205–7212.

[2] Pósfai, M. & Barabási, A. 2016 *Network science*: Citeseer.

[3] Humphries, M. D. & Gurney, K. 2008 Network 'small-world-ness': a quantitative method for determining canonical network equivalence. *PloS one.* **3**, e0002051.

[4] Watts, D. J. & Strogatz, S. H. 1998 Collective dynamics of 'small-world'networks. *Nature.* **393**, 440–442.

[5] Aprile, F., Onesto, V. & Gentile, F. 2022 The small world coefficient 4.8±1 optimizes information processing in 2D neuronal networks. *NPJ Systems Biology and Applications.* **8**, 4.

[6] Gentile, F. 2023 The effective enhancement of information in 3D small-world networks of biological neuronal cells. *Biomedical Physics & Engineering Express.* **9**, 065019.

[7] Tirinato, L., Onesto, V., Garcia-Calderon, D., Pagliari, F., Spadea, M., Seco, J. & Gentile, F. 2022 Human lung-cancer-cell radioresistance investigated through 2D network topology. *Scientific reports.* **12**, 12980.

[8] Tirinato, L., Onesto, V., Garcia-Calderon, D., Pagliari, F., Spadea, M., Seco, J. & Gentile, F. 2023 Human Cancer Cell Radiation Response Investigated through Topological Analysis of 2D Cell Networks. *Ann. Biomed. Eng.* **51**, 1859–1871.

[9] Onesto, V., Cancedda, L., Coluccio, M. L., Nanni, M., Pesce, M., Malara, N., Cesarelli, M., Di Fabrizio, E., Amato, F. & Gentile, F. 2017 Nano-topography enhances communication in neural cells networks. *Scientific reports.* **7**, 9841.

[10] Pagliari, F., Spadea, M., Montay-Gruel, P., Puspitasari-Kokko, A., Seco, J., Tirinato, L., Accardo, A., De Angelis, F. & Gentile, F. 2025 Nano-Topography Enhanced Topological-Cell-Analysis in Radiation-Therapy. *Advanced healthcare materials.*, 2405187.

[11] Marinaro, G., La Rocca, R., Toma, A., Barberio, M., Cancedda, L., Di Fabrizio, E., Decuzzi, P. & Gentile, F. 2015 Networks of neuroblastoma cells on porous silicon substrates reveal a small world topology. *Integrative Biology.* **7**, 184–197.

[12] Gentile, F. 2024 The maximum size of cell-aggregates is determined by the competition between the strain energy and the binding energy of cells. *Heliyon.* **10**.

[13] Coluccio, M. L., Onesto, V., Marinaro, G., Dell'Apa, M., De Vitis, S., Imbrogno, A., Tirinato, L., Perozziello, G., Di Fabrizio, E. & Candeloro, P. 2020 Cell theranostics on mesoporous silicon substrates. *Pharmaceutics.* **12**, 481.

[14] Waxman, B. M. 1988 Routing of multipoint connections. *IEEE J. Select. Areas Commun.* **6**, 1617–1622.
